# A Composite Maternal Healthcare Index and Its Determinants in Ethiopia: A Multilevel Analysis of Mini‐DHS 2019

**DOI:** 10.1002/puh2.70442

**Published:** 2026-11-07

**Authors:** Merga Abdissa Aga, Ding‐Geng Chen

**Affiliations:** ^1^ Department of Statistics Salale University Fiche Oromia Ethiopia; ^2^ College of Health Solutions Arizona State University Phoenix Arizona USA; ^3^ Department of Statistics University of Pretoria Pretoria South Africa

**Keywords:** Ethiopia, health inequalities, maternal healthcare utilization, multilevel ordinal logistic regression, principal component analysis

## Abstract

**Background:**

Maternal healthcare utilization is critical for maternal and neonatal health; however, many women in low‐ and middle‐income countries fail to complete the full continuum of care. Binary measures of continuity may oversimplify complex care pathways. This study developed a composite maternal healthcare utilization index and examined its associated factors in Ethiopia.

**Methods:**

A cross‐sectional analysis was conducted using the 2019 Ethiopia Mini Demographic and Health Survey. A composite index was developed using polychoric principal component analysis on the basis of antenatal care (ANC) attendance, timing of first ANC visit, place of delivery, skilled birth attendance, and postnatal care (PNC). The index was categorized into low, medium, and high utilization tertiles. Multilevel ordinal logistic regression examined individual‐ and community‐level factors associated with utilization.

**Results:**

Among 2893 women, 34.1% had low, 35.5% medium, and 30.4% high utilization. PNC contributed least to the composite index, indicating an important gap in the continuum of care. Higher education (OR = 3.16, 95% CI: 2.20–4.52), richest household wealth (OR = 2.80, 95% CI: 1.97–3.97), and urban residence (OR = 2.01, 95% CI: 1.46–2.76) were associated with higher utilization. Higher utilization was also observed in Addis Ababa (OR = 2.73, 95% CI: 1.75–4.27), SNNPR (OR = 2.28, 95% CI: 1.50–3.46), and Oromia (OR = 1.69, 95% CI: 1.12–2.54) compared with Tigray. Community‐level clustering accounted for 7.7% of the variance.

**Conclusions:**

Maternal healthcare utilization varied across socioeconomic and geographic groups, with PNC representing an important gap. Strengthening integrated maternal health services and addressing socioeconomic and geographic disparities may improve continuity of care. The composite index provides a multidimensional measure of maternal healthcare utilization.

## Graphical abstract

Using 2019 Ethiopia Mini‐DHS data, we developed a composite maternal healthcare utilization index and found substantial socioeconomic and geographic disparities. Higher education, household wealth, and urban residence were associated with higher utilization, whereas postnatal care showed the weakest contribution, highlighting an important gap in the continuum of maternal healthcare.

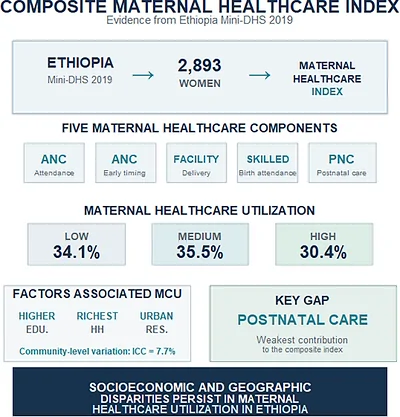

## 1 Introduction

Maternal healthcare remains a central component of global efforts to reduce preventable maternal morbidity and mortality, particularly in low‐ and middle‐income countries. Despite substantial progress under global initiatives such as the Millennium Development Goals and the Sustainable Development Goals, maternal mortality continues to be unacceptably high, with the majority of deaths occurring in sub‐Saharan Africa. Access to and utilization of essential maternal health services, including antenatal care (ANC), skilled birth attendance (SBA), and postnatal care (PNC), are critical for improving maternal and neonatal outcomes [1, 2].

In Ethiopia, considerable improvements have been observed in maternal healthcare utilization over the past two decades. However, substantial disparities persist across regions, socioeconomic groups, and urban–rural settings, limiting equitable access to essential services [3]. Nationally representative datasets, such as the Ethiopia Mini Demographic and Health Survey (EMDHS) 2019 [4], provide valuable opportunities to examine patterns and determinants of maternal healthcare utilization. Previous studies using Demographic and Health Survey data in Ethiopia have largely focused on individual components of care such as ANC attendance, institutional delivery, and PNC utilization, typically analyzed as separate binary outcomes [5, 6, 7].

Although these studies have generated important insights, they do not adequately capture the multidimensional and sequential nature of maternal healthcare utilization. Maternal care is inherently a continuum that requires consistent engagement across pregnancy, childbirth, and the postnatal period. To address this, some studies have adopted the continuum of care framework, often operationalized as the completion of recommended services [8]. However, these approaches are typically based on binary or categorical measures, which may oversimplify the complexity of service utilization and fail to capture variations in the intensity and completeness of care [9, 10].

From a methodological perspective, most existing studies rely on traditional statistical approaches, including logistic regression, survival analysis, and multilevel models, to identify determinants of maternal healthcare utilization [11, 12, 13, 14, 15]. Although these methods are effective for inference and for accounting for hierarchical data structures, the construction of a comprehensive and continuous measure of maternal healthcare utilization remains limited. In particular, there is a lack of studies that apply statistically grounded techniques such as principal component analysis (PCA) to develop composite indices that reflect the multidimensional nature of maternal healthcare utilization.

Despite these advancements, several important gaps remain. First, existing studies often rely on binary or categorical measures of maternal healthcare utilization, which may oversimplify its inherently multidimensional and sequential nature. Although prior studies have frequently relied on binary measures, this approach may result in a loss of valuable information by reducing complex care pathways into dichotomous outcomes. Second, there is limited application of statistically rigorous approaches, such as PCA, particularly polychoric PCA [16, 17, 18, 19], to construct continuous composite indices that capture variations in the intensity and completeness of care. Unlike conventional PCA based on Pearson correlations, polychoric PCA utilizes a polychoric correlation matrix estimated under a latent continuous variable framework, making it more appropriate for ordinal data. Recent methodological studies have further demonstrated that polychoric‐based approaches provide more accurate and less biased representations of underlying constructs for categorical data. Third, few studies integrate such composite measures within multilevel modeling frameworks to simultaneously examine individual‐ and community‐level determinants while accounting for the hierarchical structure of Demographic and Health Survey data.

Therefore, this study aims to address these gaps by constructing a composite index of maternal healthcare utilization using polychoric PCA within a continuum of care framework and by examining its determinants using multilevel modeling. Unlike previous studies that assess maternal health services in isolation, this approach provides a unified and continuous measure of utilization, enabling the identification of discontinuities across different stages of care. By combining statistically robust index construction with multilevel analysis, this study offers a more comprehensive and nuanced understanding of maternal healthcare utilization in Ethiopia. The findings are expected to inform targeted interventions and support evidence‐based maternal health policies.

## 2 Methods

### 2.1 Study Design and Data Source

This study employed a cross‐sectional analytical design using data from the 2019 EMDHS, conducted by the Ethiopian Public Health Institute in collaboration with ICF [4]. The EMDHS is a nationally representative survey designed to provide reliable estimates of key demographic and health indicators at national and regional levels.

The survey utilized a two‐stage stratified cluster sampling technique. In the first stage, enumeration areas (EAs) were selected using probability proportional to size. In the second stage, a fixed number of households were systematically selected within each EA. The dataset includes detailed information on women aged 15–49 years, including reproductive history and maternal healthcare utilization.

### 2.2 Study Population, Inclusion, and Exclusion Criteria

The study population consisted of women aged 15–49 years who had at least one live birth within 5 years preceding the survey.

#### 2.2.1 Inclusion Criteria

- Women aged 15–49 years.
- Women with at least one live birth in the last 5 years who and had at least one ANC.
- Women with available information on key maternal healthcare indicators.

#### 2.2.2 Exclusion Criteria

- Women with missing data on key variables, including ANC, timing of first ANC visit, place of delivery, SBA, and PNC.
- Women with inconsistent or implausible responses in maternal healthcare variables.
- Women without complete information on key explanatory variables.

After applying these criteria and data‐cleaning procedures, a final analytic sample of 2893 women was included in the analysis. Sampling weights were applied to account for the unequal probabilities of selection and nonresponse. The survey weights were normalized before analysis to preserve the national‐level representativeness of the sample. The normalized sampling weights were incorporated into the PCA used to derive the maternal healthcare utilization index and into the subsequent calculation of individual index scores.

### 2.3 Study Variables

#### 2.3.1 Outcome Variable: Composite Maternal Healthcare Index

The primary outcome of this study was a continuous composite maternal healthcare utilization index, developed to capture the multidimensional nature of maternal healthcare services.

The index was constructed using the following indicators:

- Number of ANC visits
- Timing of first ANC visit
- Place of delivery (home vs. health facility)
- SBA
- PNC

These indicators were selected on the basis of recommendations from the World Health Organization and prior empirical studies.

The index was first generated as a continuous variable and subsequently categorized into three ordered levels (low, medium, and high utilization) using tertiles for use in ordinal regression analysis.

#### 2.3.2 Explanatory Variables

The independent variables included

- Age (years)
- Educational status (no education, primary, secondary, higher)
- Marital status
- Parity (number of children ever born)
- Household wealth index
- Place of residence (urban/rural)
- Region

Community‐level variables were created by aggregating individual‐level characteristics at the cluster (EA) level and categorized using median cut‐off values.

### 2.4 Construction of the Composite Index

To construct the maternal healthcare index, PCA was applied, originally developed by Karl Pearson [16] and later formalized by Hotelling [20]. Given that the selected indicators are categorical or ordinal, a polychoric PCA approach was employed. Unlike conventional PCA based on Pearson correlations, this method relies on a polychoric correlation matrix, which estimates the association between ordinal variables under the assumption of underlying continuous latent variables. These correlations are typically obtained via maximum likelihood under a bivariate normal latent structure, making the approach more appropriate for ordinal data. Recent methodological studies have demonstrated that polychoric PCA provides more accurate and less biased estimates of the underlying structure compared to standard PCA when applied to categorical variables [21, 22].

#### 2.4.1 Data Preparation

All variables included in the analysis were recoded into numeric form to ensure compatibility with multivariate techniques. Binary variables were coded as 0 and 1. All variables were standardized (mean = 0, standard deviation = 1) to eliminate scale differences and ensure comparability across indicators.

#### 2.4.2 Assumption Checking

The suitability of the data for PCA was assessed using:

- Kaiser–Meyer–Olkin (KMO) measure (>0.6 considered adequate)
- Bartlett's test of sphericity (*p* < 0.05 indicating adequacy)

#### 2.4.3 Component Extraction

Components with eigenvalues greater than 1 were retained. The first principal component (PC1), explaining the largest variance, was selected.

#### 2.4.4 Index Construction

The composite maternal healthcare utilization index was initially treated as a continuous score. For regression analysis, the index was categorized into three ordered levels (low, medium, high) using the 33.3rd and 66.6th percentiles as cut‐off points. Tertiles were chosen to ensure balanced distribution across categories, facilitate interpretability, and maintain comparability with prior studies that classify maternal healthcare utilization into ordinal levels. Sensitivity analyses using alternative cutoffs (quartiles and continuous specification) yielded consistent results, supporting the robustness of this categorization.

### 2.5 Multilevel Ordinal Logistic Regression Analysis

Given the hierarchical structure of DHS data, multilevel ordinal regression modeling was applied to account for clustering of observations within communities [23].

A two‐level random intercept model was specified, incorporating both individual‐ and community‐level variables. Multilevel models are widely used in analyzing DHS data due to their ability to handle nested data structures [24, 25]. Model comparison was performed using intraclass correlation coefficient (ICC) and Akaike information criterion (AIC).

### 2.6 Data Management and Statistical Software

Data cleaning, recoding, and statistical analyses were conducted using R (version 4.2.1). Survey design elements, including sampling weights, clustering, and stratification, were incorporated using appropriate survey analysis procedures.

## 3 Results

Out of 8885 women of reproductive age interviewed, 3979 had a live birth within 5 years preceding the survey. Among these, 1044 women did not attend any ANC during their most recent pregnancy and were excluded from the analysis. The remaining 2935 women who had at least one ANC visit were considered. After applying sampling weights and excluding observations with missing values, 2893 women were included in the final analysis.

Among the 2893 women included in the analysis, 57.0% had four or more ANC visits, whereas 43.0% had fewer than four visits. Late initiation of ANC was reported by 57.1% of women, compared with 42.9% who initiated ANC early. Regarding delivery care, 62.6% of women delivered in a health facility, whereas 57.7% received SBA. PNC was reported by 17.0% of women, whereas 83.0% did not report receiving PNC (Table 1).

**TABLE 1:** Descriptive statistics of maternal care utilization components.

Variable	Category	Frequency	Percentage
Number of ANC visits	<4 visits	1244	43
	≥4 visits	1649	57
ANC timing	Late	1653	57.1
	Early	1240	42.9
Place of delivery	Home	1081	37.4
	Health facility	1812	62.6
Skilled birth attendance (SBA)	No	1223	42.3
	Yes	1670	57.7
Postnatal care (PNC)	No	2400	83.0
	Yes	493	17.0

Abbreviation: ANC, antenatal care.

The distribution of maternal healthcare utilization varied across several sociodemographic characteristics (Table 2). Urban women had a higher proportion in the high‐utilization category than rural women (35.3% vs. 8.5%). Similarly, women from male‐headed households had a somewhat higher proportion in the high‐utilization category than those from female‐headed households (28.0% vs. 24.1%).

**TABLE 2:** Maternal healthcare utilization by background characteristics.

Variable	Category	Low *n* (%)	Medium *n* (%)	High *n* (%)
**Residence**	Rural	508 (57.5)	300 (34.0)	75 (8.5)
	Urban	470 (23.4)	830 (41.3)	710 (35.3)
**Sex of HH**	Male	755 (32.8)	903 (39.2)	643 (28.0)
	Female	223 (37.8)	227 (38.5)	142 (24.1)
**Age group**	15–19	35 (20.7)	74 (43.8)	60 (35.5)
	20–24	223 (36.9)	241 (39.9)	140 (23.2)
	25–29	323 (34.3)	381 (40.5)	238 (25.3)
	30–34	203 (34.2)	223 (37.5)	168 (28.3)
	35–39	137 (35.5)	133 (34.5)	116 (30.1)
	40–44	48 (31.4)	56 (36.6)	49 (32.0)
	45–49	9 (20.0)	22 (48.9)	14 (31.1)
**Education (V106)**	No education	278 (22.2)	489 (39.0)	486 (38.8)
	Primary	375 (35.4)	441 (41.6)	243 (23.0)
	Secondary	174 (49.2)	138 (39.0)	42 (11.9)
	Higher	14 (6.2)	62 (27.3)	151 (66.5)
**Marital status**	Never married	8 (42.1)	8 (42.1)	3 (15.8)
	Married	892 (33.6)	1034 (39.0)	724 (27.3)
	Living together	11 (44.0)	11 (44.0)	3 (12.0)
	Widowed	8 (25.0)	12 (37.5)	12 (37.5)
	Divorced	41 (36.3)	46 (40.7)	26 (23.0)
	Separated	18 (33.3)	19 (35.2)	17 (31.5)
**Wealth index**	Poorest	304 (51.4)	204 (34.5)	84 (14.2)
	Poorer	175 (35.5)	216 (43.8)	102 (20.7)
	Middle	109 (24.0)	193 (42.5)	152 (33.5)
	Richer	147 (32.1)	210 (45.9)	101 (22.1)
	Richest	53 (5.9)	307 (34.3)	536 (59.8)
**Region**	Tigray	127 (39.6)	112 (34.9)	82 (25.5)
	Afar	56 (24.6)	74 (32.5)	98 (43.0)
	Amhara	101 (30.7)	131 (39.8)	97 (29.5)
	Oromia	71 (20.6)	163 (47.4)	110 (32.0)
	Somali	12 (14.0)	36 (41.9)	38 (44.2)
	Benishangul	91 (30.8)	131 (44.4)	73 (24.7)
	SNNPR	65 (20.0)	136 (41.8)	124 (38.2)
	Gambela	48 (18.0)	128 (47.9)	91 (34.1)
	Harari	118 (48.2)	84 (34.3)	43 (17.6)
	Addis Ababa	2 (0.9)	69 (30.7)	154 (68.4)
	Dire Dawa	27 (11.8)	66 (28.9)	135 (59.2)
**Continuous variable**	**Mean + SD**	**Minimum**	**Maximum**	
Number of children ever born	3.43 + 2.32	1	15	

The distribution of utilization across age groups did not show a clear monotonic pattern. The proportion of women in the high‐utilization category ranged from 23.2% among those aged 20–24 years to 35.5% among those aged 15–19 years. Educational attainment showed a marked gradient: The proportion in the high‐utilization category was 38.8% among women with no formal education, 23.0% among those with primary education, 11.9% among those with secondary education, and 66.5% among those with higher education. These descriptive differences were further examined using multilevel ordinal logistic regression.

Utilization also varied across household wealth categories. The proportion in the high‐utilization category was highest among women in the richest households (59.8%) and lowest among those in the poorer households (20.7%), whereas 14.2% of women in the poorest households were in the high‐utilization category. Regional variation was also observed. The highest proportions of women in the high‐utilization category were observed in Addis Ababa (68.4%) and Dire Dawa (59.2%), whereas lower proportions were observed in Harari (17.6%) and Tigray (25.5%). The mean number of children ever born was 3.43 (SD = 2.32), with values ranging from 1 to 15 (Table 2).

Overall, maternal care utilization in Ethiopia is strongly associated with place of residence, education level, wealth status, and region, with urban centers such as Addis Ababa and Dire Dawa demonstrating significantly higher utilization compared to rural and less developed regions.

### 3.1 Construction of Maternal Healthcare Utilization Index

A polychoric PCA was employed to construct the maternal healthcare utilization index, as the indicators are ordinal and binary in nature. This approach uses polychoric correlations, which are more appropriate than Pearson correlations for categorical data.

The PCA results indicate that all maternal healthcare service indicators load positively on the PC1, suggesting that they jointly measure a common underlying construct, maternal healthcare utilization. Among the indicators, ANC visits (loading = 0.779) and timing of the first ANC visit (loading = 0.696) contribute most strongly to the index. This implies that early initiation and adequate frequency of ANC are the most influential components in defining overall maternal healthcare utilization in the study population. Facility delivery (0.602) and SBA (0.636) also show substantial contributions, reflecting the importance of institutional and professional care during childbirth in shaping the composite index (Table 3). In contrast, PNC has a relatively lower loading (0.335), indicating that it contributes less to the overall variability captured by PC1. This may suggest that PNC utilization is less consistent or less strongly correlated with other maternal health services, potentially reflecting gaps in postnatal service uptake.

**TABLE 3:** Principal component analysis (PCA) results for maternal healthcare utilization index.

Variable	Factor loading (PC1)
ANC visits	0.779
Timing of first ANC	0.696
Facility delivery	0.602
Skilled birth attendance	0.636
Postnatal care (PNC)	0.335

Abbreviations: ANC, antenatal care; PC1, first principal component.

The scree plot shows a clear elbow after the first component, indicating that the PC1 explains the largest proportion of variance in the data. The first component has an eigenvalue greater than 1, satisfying the Kaiser criterion, whereas all subsequent components have eigenvalues equal to or less than 1 (Figure 1).

**FIGURE 1:**
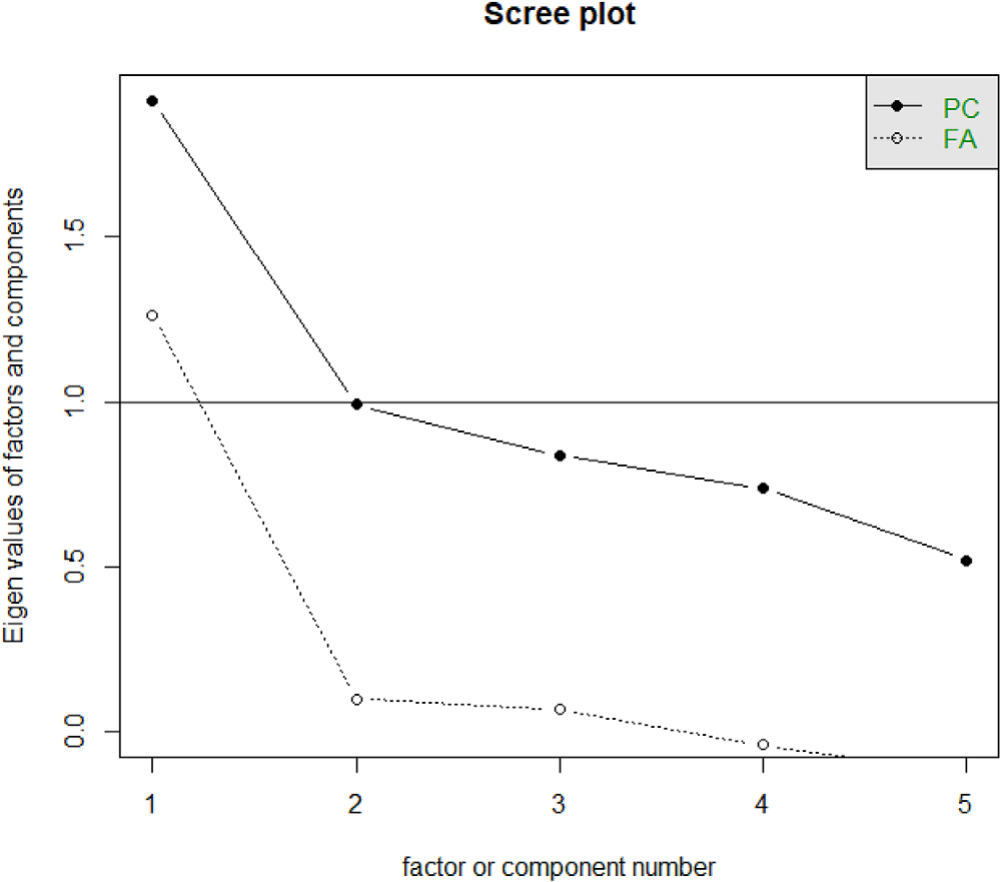
Scree plot for principal component (PC) and factor analysis (FA). Scree plot displaying the eigenvalues of PC and FA for maternal healthcare utilization indicators. The horizontal line at eigenvalue = 1 represents the Kaiser criterion for component retention.

Both PC and FA results consistently suggest that only one component/factor should be retained. The sharp decline from the first to the second component indicates that additional components contribute minimal explanatory power.

This finding supports the use of a single composite index to represent maternal healthcare utilization, reinforcing the validity of combining ANC visits, ANC timing, facility delivery, SBA, and PNC into one summary measure.

This pattern supports the idea that maternal healthcare utilization is a multidimensional but interconnected construct. A moderate positive correlation is observed between ANC visits and the timing of the first ANC visit, suggesting that women who initiate care early are more likely to complete a higher number of visits. This reflects adherence to recommended maternal care practices (Figure 2).

The heatmap of polychoric correlations reveals that all maternal healthcare utilization indicators are positively associated, indicating that these services tend to be used jointly rather than independently.

**FIGURE 2:**
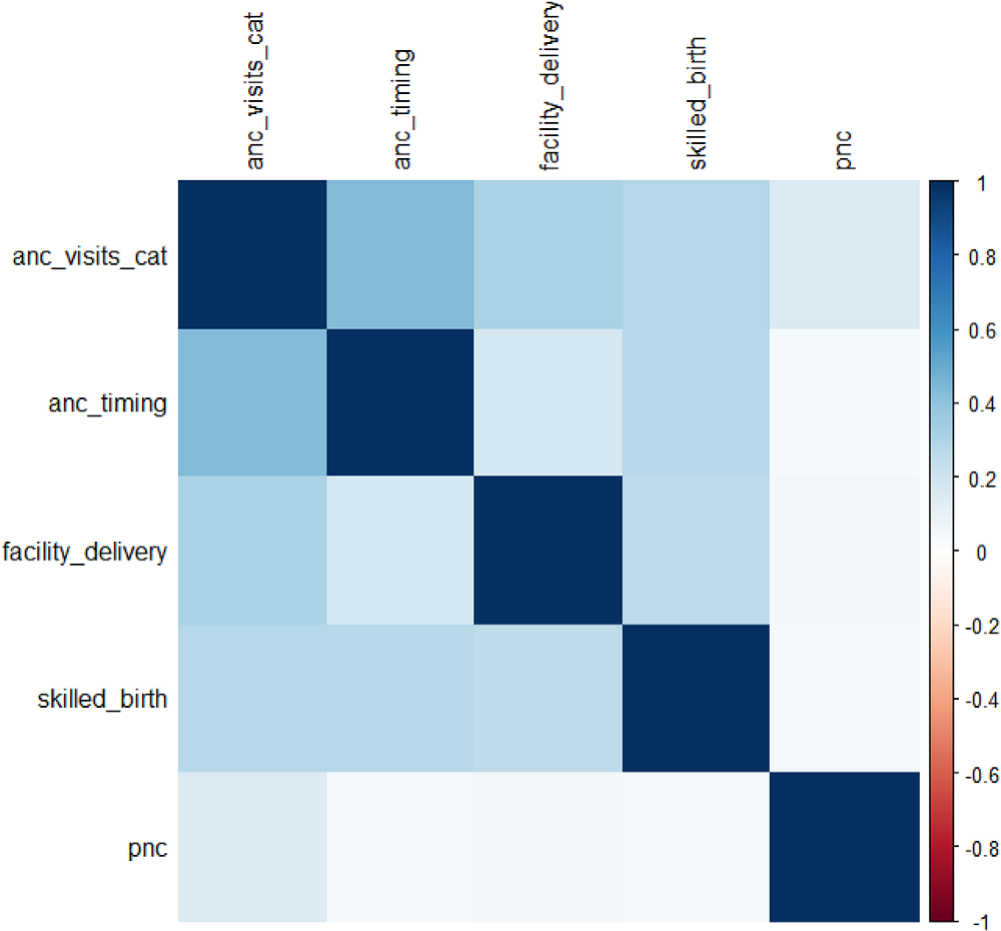
Polychoric correlation matrix of maternal healthcare utilization indicators. ANC, antenatal care; PNC, postnatal care.

Facility‐based delivery and SBA exhibit a strong positive correlation, which is expected given that both services are typically provided simultaneously within health facilities. This strong association reinforces their joint contribution to safe delivery practices. Furthermore, ANC‐related indicators (number of visits and timing) show moderate correlations with delivery care services, supporting the continuum of care framework, where engagement during pregnancy increases the likelihood of utilizing skilled care during childbirth (Figure 2).

In contrast, PNC demonstrates relatively weaker correlations with other maternal healthcare components. This suggests that PNC utilization may depend on different behavioral, cultural, or accessibility factors and may not be as consistently utilized even among women who receive adequate antenatal and delivery care.

The overall pattern of positive and meaningful correlations among the indicators provides empirical justification for combining them into a composite maternal healthcare utilization index. The absence of strong negative or negligible correlations indicates that these variables measure related aspects of a common latent construct, supporting the appropriateness of using PCA.

The KMO measure of sampling adequacy was 0.76, indicating good sampling adequacy for PCA. Bartlett's test of sphericity was statistically significant (*χ* ^2^ = 1430.67, df = 10, *p* < 0.001), rejecting the null hypothesis that the correlation matrix is an identity matrix. These findings indicate that the maternal healthcare indicators were sufficiently intercorrelated to support the application of PCA (Table 4).

**TABLE 4:** Principal component analysis (PCA) adequacy and variance explained.

Measure	Value
KMO (overall)	0.76
Bartlett's test (*χ* ^2^)	1430.67
Degrees of freedom	10
*p* value	<0.001
Eigenvalue (PC1)	1.97
Variance explained (%)	39.4

Abbreviations: KMO, Kaiser–Meyer–Olkin; PC1, first principal component.

The PC1 had an eigenvalue of 1.97, exceeding the commonly used Kaiser criterion of 1. PC1 explained 39.4% of the total variance across the five indicators. This component was therefore retained as the basis for constructing the continuous maternal healthcare index.

### 3.2 Distribution of Maternal Healthcare Utilization

The distribution of maternal healthcare utilization shows a relatively balanced pattern across categories. Approximately 34.1% of women fall into the low utilization category, 35.5% into the medium category, and 30.4% into the high utilization category.

Although a considerable proportion of women utilize some maternal health services, less than one‐third achieve high utilization. This indicates persistent gaps in completing the full continuum of maternal care, particularly among those in the low utilization group.

Bar plot illustrating the distribution of maternal healthcare utilization levels categorized as low, medium, and high based on the composite index. The color scheme reflects the level of utilization, with red indicating low utilization, yellow representing medium utilization, and green indicating high utilization. Percentage labels are displayed above each bar to show the proportion of women in each category (Figure 3).

**FIGURE 3:**
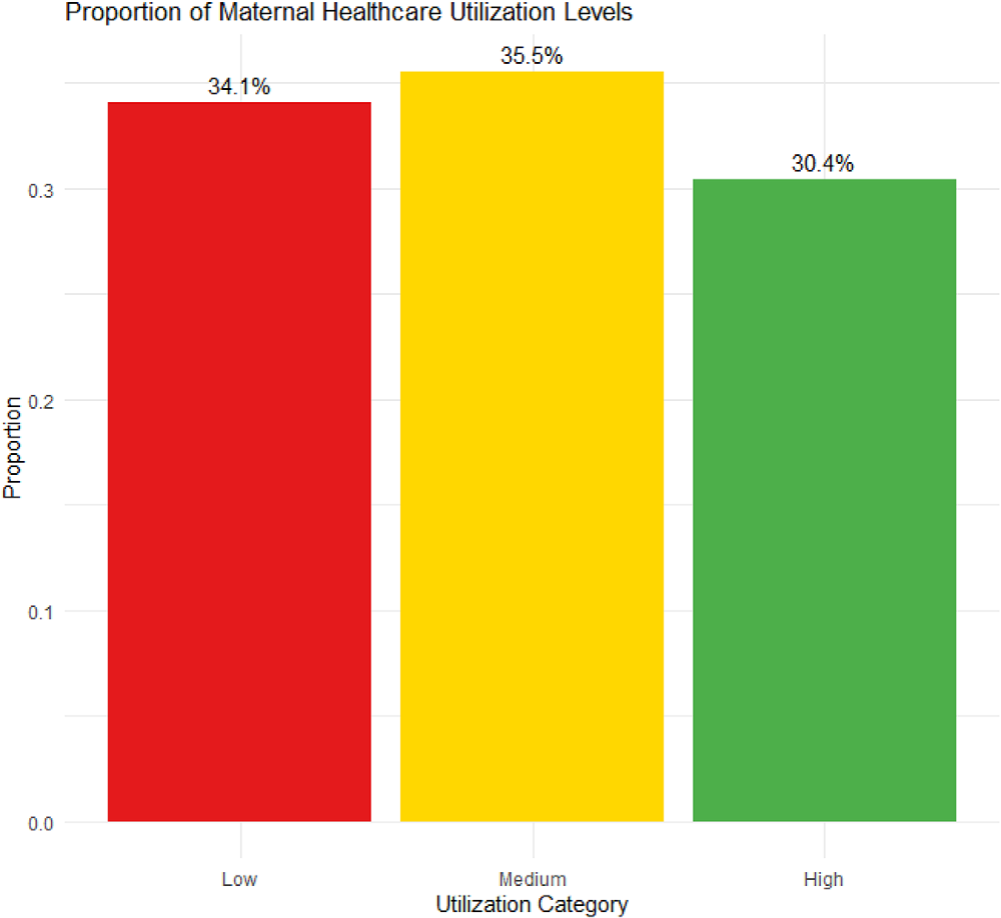
Proportion of maternal healthcare utilization levels.

This distribution suggests that although a considerable proportion of women access some maternal healthcare services, optimal utilization remains limited, as less than one‐third achieve high utilization. The presence of a substantial low‐utilization group highlights persistent gaps in accessing the full continuum of maternal care services.

### 3.3 Multilevel Ordinal Logistic Regression Results

The results presented in Table 5 indicate that the multilevel ordinal logistic regression model was fitted to examine the factors associated with the maternal care utilization index. The model accounted for the hierarchical structure of the data, with individuals nested within clusters, and estimated the effects of both individual‐ and community‐level variables on the ordered levels of maternal care utilization.

**TABLE 5:** Multilevel ordinal logistic regression results.

Variable	Category	*β*	SE	OR (95% CI)	*p* value
Household head	Male (ref.)				
	Female	−0.157	0.111	0.85 (0.69–1.06)	0.158
Age group	15–19 (ref.)				
	20–24	−0.311	0.178	0.73 (0.52–1.04)	0.081
	25–29	−0.352	0.179	0.70 (0.50–1.00)	0.049
	30–34	−0.546	0.198	0.58 (0.39–0.85)	0.006
	35–39	−0.669	0.221	0.51 (0.33–0.79)	0.003
	40–44	−0.628	0.269	0.53 (0.31–0.90)	0.020
	45–49	−0.620	0.369	0.54 (0.26–1.11)	0.093
Education	None (ref.)				
	Primary	0.586	0.097	1.80 (1.49–2.17)	<0.001
	Secondary	0.830	0.145	2.29 (1.73–3.05)	<0.001
	Higher	1.149	0.183	3.16 (2.20–4.52)	<0.001
Marital status	Unmarried (ref.)				
	Married	0.425	0.481	1.53 (0.60–3.93)	0.377
	Living together	1.241	0.553	3.46 (1.17–10.23)	0.025
	Widowed	0.326	0.606	1.39 (0.42–4.54)	0.591
	Divorced	0.412	0.510	1.51 (0.56–4.10)	0.419
	Separated	−0.279	0.627	0.76 (0.22–2.59)	0.657
Children ever born	Number	0.033	0.025	1.03 (0.98–1.09)	0.195
Wealth index	Poorest (ref.)				
	Poorer	0.548	0.133	1.73 (1.33–2.24)	<0.001
	Middle	0.542	0.140	1.72 (1.31–2.26)	<0.001
	Richer	0.854	0.145	2.35 (1.77–3.12)	<0.001
	Richest	1.029	0.179	2.80 (1.97–3.97)	<0.001
Residence	Rural (ref.)				
	Urban	0.697	0.162	2.01 (1.46–2.76)	<0.001
Region	Tigray (ref.)				
	Afar	0.401	0.244	1.49 (0.93–2.41)	0.101
	Amhara	−0.003	0.210	1.00 (0.66–1.50)	0.910
	Oromia	0.522	0.209	1.69 (1.12–2.54)	0.013
	Somali	0.897	0.314	2.45 (1.33–4.54)	0.004
	Benishangul	−0.059	0.222	0.94 (0.61–1.46)	0.790
	SNNPR	0.825	0.213	2.28 (1.50–3.46)	<0.001
	Gambela	0.192	0.265	1.21 (0.72–2.04)	0.469
	Harari	0.355	0.236	1.43 (0.90–2.26)	0.132
	Addis Ababa	1.004	0.228	2.73 (1.75–4.27)	<0.001
	Dire Dawa	0.507	0.246	1.66 (1.03–2.69)	0.039

The multilevel ordinal logistic regression analysis identified several demographic, socioeconomic, and geographic factors associated with the level of maternal healthcare utilization (Table 5). The odds ratios represent the odds of being in a higher maternal healthcare utilization category relative to the specified reference category.

Age group was significantly associated with maternal healthcare utilization. Compared with women aged 15–19 years, women aged 25–29 years had lower odds of being in a higher utilization category (OR = 0.70, 95% CI: 0.50–1.00; *p* = 0.049). The odds were further reduced among women aged 30–34 years (OR = 0.58, 95% CI: 0.39–0.85; *p* = 0.006), 35–39 years (OR = 0.51, 95% CI: 0.33–0.79; *p* = 0.003), and 40–44 years (OR = 0.53, 95% CI: 0.31–0.90; *p* = 0.020). However, the associations for women aged 20–24 years (OR = 0.73, 95% CI: 0.52–1.04; *p* = 0.081) and 45–49 years (OR = 0.54, 95% CI: 0.26–1.11; *p* = 0.093) were not statistically significant. Thus, the evidence does not support a uniform association across all older age groups.

Educational attainment showed a strong positive association with maternal healthcare utilization. Compared with women who had no formal education, those with primary education had higher odds of being in a higher utilization category (OR = 1.80, 95% CI: 1.49–2.17; *p* < 0.001). The association was stronger among women with secondary education (OR = 2.29, 95% CI: 1.73–3.05; *p* < 0.001) and higher education (OR = 3.16, 95% CI: 2.20–4.52; *p* < 0.001). These findings indicate a positive gradient between educational attainment and maternal healthcare utilization.

Household wealth was also positively associated with utilization. Compared with women from the poorest households, women in the poorer (OR = 1.73, 95% CI: 1.33–2.24), middle (OR = 1.72, 95% CI: 1.31–2.26), richer (OR = 2.35, 95% CI: 1.77–3.12), and richest (OR = 2.80, 95% CI: 1.97–3.97) categories had significantly higher odds of being in a higher utilization category (all *p* < 0.001). The magnitude of the associations was generally greater among women in the richer and richest wealth categories, indicating substantial socioeconomic differences in maternal healthcare utilization.

Place of residence was significantly associated with utilization. Compared with women living in rural areas, urban women had approximately twice the odds of being in a higher utilization category (OR = 2.01, 95% CI: 1.46–2.76; *p* < 0.001).

There was also significant geographic variation in maternal healthcare utilization. Using Tigray as the reference region, women in Oromia had higher odds of being in a higher utilization category (OR = 1.69, 95% CI: 1.12–2.54; *p* = 0.013), as did women in Somali (OR = 2.45, 95% CI: 1.33–4.54; *p* = 0.004), SNNPR (OR = 2.28, 95% CI: 1.50–3.46; *p* < 0.001), Addis Ababa (OR = 2.73, 95% CI: 1.75–4.27; *p* < 0.001), and Dire Dawa (OR = 1.66, 95% CI: 1.03–2.69; *p* = 0.039). In contrast, the associations for Afar, Amhara, Benishangul‐Gumuz, Gambela, and Harari were not statistically significant compared with Tigray.

Marital status was significantly associated with utilization only among women living together. Compared with unmarried women, women who were living together had higher odds of being in a higher utilization category (OR = 3.46, 95% CI: 1.17–10.23; *p* = 0.025). No statistically significant associations were observed among married (OR = 1.53, 95% CI: 0.60–3.93; *p* = 0.377), widowed (OR = 1.39, 95% CI: 0.42–4.54; *p* = 0.591), divorced (OR = 1.51, 95% CI: 0.56–4.10; *p* = 0.419), or separated women (OR = 0.76, 95% CI: 0.22–2.59; *p* = 0.657).

The comparison between the null and final multilevel ordinal logistic regression models demonstrates a clear improvement in model fit after the inclusion of explanatory variables.

The log‐likelihood increases (from −2845.72 to −2789.41), and the AIC decreases (from 5695.44 to 5646.81) in the final model, indicating a better fit to the data. This confirms that the inclusion of individual‐ and community‐level predictors significantly enhances the model's explanatory power (Table 6). At the cluster level, the variance decreases from 0.342 in the null model to 0.275 in the final model, with the corresponding standard deviation declining from 0.585 to 0.524. This reduction suggests that a substantial portion of the between‐cluster variation in maternal healthcare utilization is explained by the covariates included in the model.

**TABLE 6:** Comparison of null and final multilevel ordinal logistic models.

Measure	Null model	Final model
Log‐likelihood	−2845.72	−2789.41
AIC	5695.44	5646.81
Cluster variance (V021)	0.342	0.275
Standard deviation	0.585	0.524
ICC (%)	9.4	7.7
Number of clusters	303	303

Abbreviations: AIC, Akaike information criterion; ICC, Intra‐class correlation coefficient.

Similarly, the ICC declines from 9.4% to 7.7%, indicating that the proportion of total variation attributable to differences between clusters is reduced after adjusting for observed characteristics. However, the remaining ICC of 7.7% suggests that unobserved community‐level factors continue to play a meaningful role in influencing maternal healthcare utilization (Table 6).

The persistence of non‐negligible cluster‐level variation even after adjustment highlights the importance of contextual and community‐level influences, such as healthcare infrastructure, cultural practices, and regional accessibility, which are not fully captured by the included variables.

### 3.4 Sensitivity Analysis

Sensitivity analyses were conducted to assess whether the findings were sensitive to the categorization and treatment of the maternal healthcare utilization index. When the index was categorized into quartiles rather than tertiles, the direction and overall pattern of the associations were generally consistent with those observed in the primary multilevel ordinal logistic regression model. Similarly, analysis of the maternal healthcare utilization index as a continuous outcome produced results that were consistent with the primary analysis. The principal associations involving educational attainment, household wealth, place of residence, and geographic characteristics remained evident across the alternative specifications. Overall, these analyses indicated that the main findings were robust to alternative specifications of the maternal healthcare utilization index (Table S1).

## 4 Discussion

This study developed a composite index of maternal healthcare utilization using polychoric PCA and examined its determinants within a continuum of care framework. The findings reveal substantial gaps and inequalities in maternal health service utilization across different stages of care in Ethiopia.

The results confirm that maternal healthcare utilization is a continuum encompassing ANC, skilled delivery, and PNC. Although all components contributed positively to the index, PNC demonstrated a relatively weak contribution, indicating a critical discontinuity in the maternal care pathway. This suggests that although many women initiate care during pregnancy, a considerable proportion fail to continue through to the postnatal period. Recent evidence continues to highlight PNC as the weakest link in the continuum of maternal healthcare in low‐ and middle‐income countries [1, 2].

From a methodological perspective, women with no utilization of maternal healthcare services were classified in the lowest category of the index, as they received the minimum scores across all indicators. Although this approach is consistent with previous applications of PCA, it may obscure heterogeneity within the low‐utilization group, particularly between women who received no care and those with partial service use. Additionally, although SBA and place of delivery are closely related, they capture distinct dimensions of care provider competence and service location and were therefore retained as separate indicators. These considerations should be taken into account when interpreting the findings.

The observed discontinuity is consistent with previous evidence from Ethiopia, where completion of the continuum of care remains low. Studies have reported substantial dropouts after delivery, with only a small proportion of women completing all stages of maternal healthcare [26, 27]. Similar patterns have been documented in other low‐ and middle‐income countries, including Nepal and Indonesia, where PNC remains the least utilized component despite improvements in antenatal and delivery services [28, 29]. These findings underscore that PNC represents a critical gap in maternal healthcare systems.

The relatively modest internal coherence of the composite index reflects the multidimensional and sequential nature of maternal healthcare utilization. Unlike psychometric constructs, maternal healthcare services involve a series of behaviors influenced by different contextual factors at each stage. As a result, women may utilize some services while missing others, leading to discontinuities across the care pathway. In this context, PCA remains an appropriate approach, as it captures shared variation among indicators without imposing strict assumptions of unidimensionality [30].

The distribution of utilization levels further indicates that only a limited proportion of women achieve high utilization across all stages of care. This aligns with national evidence from Ethiopia, where full continuum utilization remains low despite improvements in individual service coverage [31]. Similar trends across sub‐Saharan Africa highlight persistent structural barriers, including poverty, limited healthcare access, and sociocultural constraints [32].

The analysis identified several key determinants of maternal healthcare utilization. Maternal education emerged as a strong positive predictor, suggesting that educated women are more likely to understand the importance of maternal healthcare and effectively navigate available services. This finding is consistent with evidence from other low‐ and middle‐income settings [33]. Household wealth also showed a clear positive gradient, underscoring the persistent role of socioeconomic inequality in shaping access to care [34].

Place of residence was another significant determinant, with urban women demonstrating substantially higher utilization than rural women. This disparity likely reflects differences in healthcare infrastructure, accessibility, and availability of skilled providers, consistent with findings from other developing countries [35]. Furthermore, early initiation of ANC was associated with higher overall utilization, highlighting the importance of timely entry into the healthcare system for ensuring continuity of care [29].

The study also revealed significant community‐level variation, indicating that contextual factors beyond individual characteristics influence maternal healthcare utilization. Regional disparities in infrastructure, cultural practices, and service availability likely contribute to these differences. This finding reinforces the importance of incorporating multilevel approaches in maternal health research [26].

### 4.1 Policy Implications

These findings have important policy implications. Strengthening PNC services is critical to ensuring continuity across the maternal healthcare continuum. Interventions should prioritize not only access but also sustained engagement through strategies such as community‐based follow‐up, postnatal home visits, and targeted awareness campaigns.

Efforts to reduce socioeconomic inequalities, particularly through improving female education and expanding financial protection mechanisms, are essential for enhancing maternal healthcare utilization. Addressing rural–urban disparities by improving healthcare infrastructure, transportation systems, and workforce distribution in underserved areas is equally important. In addition, region‐specific strategies are needed to address contextual barriers and ensure equitable access to maternal healthcare services.

At the global level, improving maternal healthcare utilization is essential for achieving the targets of the Sustainable Development Goals, particularly those related to reducing maternal mortality.

### 4.2 Limitation of the Study

This study has several limitations. First, the cross‐sectional design limits the ability to establish temporal or causal relationships between the explanatory factors and maternal healthcare utilization. Second, the reliance on self‐reported information may have introduced recall and reporting bias, particularly for measures of maternal healthcare utilization. Third, although polychoric PCA was used to construct a composite index from multiple categorical healthcare indicators, the resulting index may not fully capture the multidimensional complexity of maternal healthcare utilization or unobserved aspects of care. Fourth, the continuous PCA‐derived index was categorized into tertiles for the primary multilevel ordinal logistic regression analysis. Although this facilitated interpretation, categorization may have resulted in some loss of information and within‐category variation. Sensitivity analyses using quartile categorization and the index as a continuous outcome produced broadly consistent patterns of associations, supporting the robustness of the main findings. Finally, potentially relevant factors, including cultural beliefs, perceived quality of care, women's preferences, and health‐system characteristics, were not available in the dataset and therefore could not be incorporated into the analysis. Future studies incorporating these contextual and health‐system factors and retaining the utilization index as a continuous measure may provide a more comprehensive assessment of maternal healthcare utilization.

## 5 Conclusions

This study developed a composite index of maternal healthcare utilization within a continuum of care framework and identified its key determinants. The findings indicate that although ANC and facility‐based delivery have improved, substantial gaps remain in ensuring continuity of care, particularly during the postnatal period.

The relatively weak contribution of PNC highlights a critical discontinuity in maternal healthcare utilization, suggesting that many women do not complete the full continuum of care. This underscores the need to move beyond isolated service improvements toward integrated strategies that promote sustained engagement across pregnancy, childbirth, and the postpartum period.

Maternal healthcare utilization is strongly influenced by socioeconomic and demographic factors, including education, household wealth, place of residence, and timing of ANC initiation. Persistent inequalities, especially between urban and rural populations, continue to limit equitable access to essential maternal health services.

Furthermore, the observed community‐level variation emphasizes the importance of contextual factors, such as regional disparities, healthcare infrastructure, and service availability, in shaping utilization patterns.

Overall, improving maternal health outcomes requires a comprehensive, equity‐focused approach that strengthens continuity of care. Priority should be given to enhancing PNC services, reducing socioeconomic and geographic disparities, and expanding access to quality maternal healthcare in underserved areas. Promoting early initiation of ANC and ensuring progression through all stages of care are essential for achieving meaningful improvements.

At a broader level, addressing these gaps is critical for accelerating progress toward global maternal health targets, particularly the reduction of preventable maternal mortality in low‐ and middle‐income countries.

## Author Contributions

**Merga Abdissa Aga** mainly designed the research and led the overall conceptualization of the study. Merga Abdissa Aga and **Ding‐Geng Chen** contributed to the study design, data extraction, descriptive and analytical data analysis, interpretation of results, and drafting of the manuscript. Merga Abdissa Aga also led data cleaning and preprocessing, preparation of figures and tables, data visualization, and critical revision of the manuscript. Ding‐Geng Chen reviewed and edited the manuscript. All authors thoroughly reviewed the manuscript and approved the final version for submission.

## Funding

The authors have nothing to report.

## Ethics Statement

This study used secondary data from the Mini Demographic and Health Surveys (DHS) Program. Formal permission to access and use the dataset was obtained. The MDHS surveys were conducted in accordance with ethical standards and approved by relevant institutional review boards. The data are anonymized and publicly available; therefore, no additional ethical approval was required.

## Consent

The study used secondary data from the Ethiopia Mini Demographic and Health Survey (Mini‐DHS) 2019. Informed consent was obtained from all participants by the DHS program prior to data collection. Permission to use the dataset was obtained from the DHS Program.

## Conflicts of Interest

The authors declare no conflicts of interest.

## Supporting information




**Supporting File 1**: puh270442‐sup‐0001‐S1.docx.

## Data Availability

The data that support the findings of this study are available from the DHS Program database (https://dhsprogram.com) upon reasonable request and permission.
