# Supplementary material for: A Composite Maternal Healthcare Index and Its Determinants in Ethiopia: A Multilevel Analysis of Mini‐DHS 2019
Source: Public Health Chall. 2026 Nov 7;5(4):e70442. doi: 10.1002/puh2.70442 (PMC13744617; doi:10.1002/puh2.70442)
Supplement: Supplementary file 1 [file PUH2-5-e70442-s001.docx]

**Supplementary Table S1:** Sensitivity analyses of factors associated with maternal healthcare utilization using alternative specifications of the utilization index

| **Variable** | **Primary: Tertiles β** | **Quartiles β** | **Continuous β** |
| --- | --- | --- | --- |
| **Household head** |  |  |  |
| Male (ref.) | — | — | — |
| Female | −0.157 | −0.142 | −0.061 |
| **Age group** |  |  |  |
| 15–19 (ref.) | — | — | — |
| 20–24 | −0.311 | −0.286 | −0.118 |
| 25–29 | −0.352 | −0.341 | −0.137 |
| 30–34 | −0.546 | −0.512 | −0.205 |
| 35–39 | −0.669 | −0.628 | −0.247 |
| 40–44 | −0.628 | −0.601 | −0.231 |
| 45–49 | −0.620 | −0.574 | −0.218 |
| **Education** |  |  |  |
| None (ref.) | — | — | — |
| Primary | 0.586 | 0.571 | 0.221 |
| Secondary | 0.830 | 0.798 | 0.306 |
| Higher | 1.149 | 1.102 | 0.421 |
| **Marital status** |  |  |  |
| Unmarried (ref.) | — | — | — |
| Married | 0.425 | 0.397 | 0.152 |
| Living together | 1.241 | 1.183 | 0.451 |
| Widowed | 0.326 | 0.301 | 0.117 |
| Divorced | 0.412 | 0.386 | 0.148 |
| Separated | −0.279 | −0.254 | −0.098 |
| **Children ever born** |  |  |  |
| Number | 0.033 | 0.030 | 0.012 |
| **Wealth index** |  |  |  |
| Poorest (ref.) | — | — | — |
| Poorer | 0.548 | 0.526 | 0.204 |
| Middle | 0.542 | 0.519 | 0.199 |
| Richer | 0.854 | 0.821 | 0.315 |
| Richest | 1.029 | 0.987 | 0.379 |
| **Residence** |  |  |  |
| Rural (ref.) | — | — | — |
| Urban | 0.697 | 0.671 | 0.258 |
| **Region** |  |  |  |
| Tigray (ref.) | — | — | — |
| Afar | 0.401 | 0.374 | 0.143 |
| Amhara | −0.003 | −0.011 | −0.004 |
| Oromia | 0.522 | 0.496 | 0.190 |
| Somali | 0.897 | 0.851 | 0.327 |
| Benishangul | −0.059 | −0.051 | −0.020 |
| SNNPR | 0.825 | 0.786 | 0.302 |
| Gambela | 0.192 | 0.176 | 0.068 |
| Harari | 0.355 | 0.331 | 0.127 |
| Addis Ababa | 1.004 | 0.958 | 0.368 |
| Dire Dawa | 0.507 | 0.481 | 0.185 |
